# Extended Release of Bupivacaine from Temperature-Responsive PNDJ Hydrogels Improves Postoperative Weight-Bearing in Rabbits Following Knee Surgery

**DOI:** 10.3390/ph17070879

**Published:** 2024-07-03

**Authors:** Derek J. Overstreet, Gabriel Zdrale, Alex C. McLaren

**Affiliations:** 1 School of Biological & Health Systems Engineering, Arizona State University, Tempe, AZ 85287, USA; 2 Sonoran Biosciences, Tempe, AZ 85284, USA; 3 Department of Orthopaedic Surgery, University of Arizona College of Medicine, Phoenix, AZ 85004, USA

**Keywords:** hydrogel, injectable, non-opioid, acute pain, local anesthetic, extended release, postoperative pain, knee, orthopaedic surgery

## Abstract

Effective treatment of postoperative pain lasting for multiple days without opioids is an important clinical need. We previously reported analgesia lasting up to 96 h in a porcine soft tissue model of postoperative pain using SBG004, an extended-release formulation of bupivacaine based on the temperature-responsive polymer poly(*N*-isopropylacrylamide-*co*-dimethylbutyrolactone acrylamide-*co*-Jeffamine M-1000 acrylamide) [PNDJ]. Orthopaedic surgical sites such as the knee can involve complex sensory innervation which presents a distinct challenge to local anesthetic delivery. The purpose of this work was to evaluate the pharmacokinetics and efficacy of SBG004 in an orthopaedic surgical model in comparison to currently available local anesthetics. Pharmacokinetics following periarticular (PA) or intraarticular (IA) injection of SBG004 were compared against liposomal bupivacaine (Lip-Bupi) PA in New Zealand White rabbits (all doses 14.5 mg/kg). Analgesic efficacy of SBG004 (IA, PA, or IA + PA), three active comparators, and saline was evaluated following knee surgery in New Zealand White rabbits. Analgesia was assessed via weight-bearing on the operated limb during spontaneous large steps in video recordings. Systemic bupivacaine exposure lasted at least 7 days for SBG004 PA, 4 days for SBG004 IA, and 2 days for Lip-Bupi PA. In the analgesia study, weight-bearing in all active groups except SBG004 IA was more frequent versus saline through 8 h postoperatively (*p* < 0.05). Only SBG004 IA + PA resulted in a higher proportion of weight-bearing rabbits at 24 h versus saline (6/7 versus 2/10, *p* = 0.015). Analysis of pooled data from 24–72 h showed significantly greater frequency of weight-bearing in rabbits receiving SBG004 IA + PA (71%) versus saline (37%), ropivacaine cocktail (41%), and Lip-Bupi PA (36%). The results indicate that the release profile from SBG004 PA or IA coincides reasonably with the time course of postoperative pain, and SBG004 may produce longer duration of analgesia than local anesthetics currently used in knee surgery, including during the period of 24–72 h recognized as a target for extended-release local anesthetics.

## 1. Introduction

Total knee arthroplasty (TKA) is one of the most common surgical procedures associated with moderate to severe postoperative pain. Pain following TKA is conducted along extensive sensory nerves distributed throughout the tissues surrounding the joint. Multimodal analgesic regimens are the standard for perioperative pain management in TKA [1]. A common component of multimodal analgesic regimens in TKA is global periarticular injection (tissue infiltration) of a solution containing a local anesthetic, usually ropivacaine or bupivacaine, and optionally other analgesics [1,2]. Local anesthetics can reduce or eliminate the sensation of pain for up to 6–8 h when administered by tissue infiltration, but patients often experience moderate to severe pain after this time [3,4,5,6]. There are two extended-release local anesthetics (ERLAs) currently approved by the FDA which are indicated for use in TKA. These products, liposomal bupivacaine (Lip-Bupi, trade name Exparel, Pacira Biosciences, Parsippany, NJ, USA) and polyorthoester bupivacaine–meloxicam (POE-Bupi-Mel, trade name Zynrelef, Heron Therapeutics, San Diego, CA, USA), are intended to extend the duration of analgesia for up to 72 h. However, randomized controlled trials generally indicate that these ERLAs provide minimal additional analgesia versus plain local anesthetics in TKA as measured by reduction in pain scores and opioid consumption [6,7,8,9,10,11,12].

We have previously described extended release of bupivacaine from hydrogels based on the temperature-responsive polymer poly(*N*-isopropylacrylamide-*co*-dimethylbutyrolactone acrylamide-*co*-Jeffamine M-1000 acrylamide) [PNDJ] [13]. At room temperature, an aqueous solution of PNDJ can be mixed with bupivacaine suspension. The resulting suspension is injectable. It rapidly thickens to form a soft, semi-solid gel on warming from contact with tissue as it approaches body temperature. Potential advantages of using PNDJ gel as a vehicle for bupivacaine delivery include a suitable release profile, higher drug loading than current ERLAs, compatibility with injection to precisely distribute the desired dose, and retention at the dosing site without dilution. We observed no adverse effects following subcutaneous administration of SBG004 in rabbits (16.5 mg bupivacaine/kg), and gel was histologically absent at 49 days [13]. A GLP toxicology study in minipigs found no adverse findings following subcutaneous and intraperitoneal injection of PNDJ alone [14]. We previously reported that a formulation of PNDJ containing 4 wt% bupivacaine (abbreviated as SBG004) released bupivacaine for over 7 days following subcutaneous injection in rabbits and reduced mechanical allodynia in a porcine skin and muscle incision model for 96 h [13].

In this work, we sought to evaluate the feasibility of SBG004 in a procedure similar to TKA to complement the previously reported data in soft tissue surgery. TKA involves pain originating from different tissue types and a different pattern of sensory innervation compared to soft tissue procedures. Pharmacokinetics of released bupivacaine from SBG004 were measured when applied by periarticular (PA) or intraarticular (IA) injection in comparison to periarticular Lip-Bupi to estimate the in vivo release profile. Lip-Bupi was used as the comparator because it is the leading ERLA in clinical use and is injectable at the same dose. We also compared the analgesic efficacy of SBG004 with Lip-Bupi, POE-Bupi-Mel, and a ropivacaine-based local anesthetic cocktail (Ropi) following knee surgery in rabbits as indicated by weight-bearing during spontaneous ambulation.

## 2. Results

### 2.1. PNDJ and SBG004 Characterization

PNDJ was successfully synthesized with repeat unit content of 79.89: 18.68: 1.43 mol% for N-isopropyacrylamide (NIPAAm), dimethyl-butyrolactone acrylamide (DBLAAm), and Jeffamine M-1000 acrylamide (JAAm) units, respectively. Weight-average molecular weight was 35,000 g/mol. As reported previously, SBG004 had viscosity of 2.10 Pa*s at 5 °C, gelation temperature of 19 °C, and in vitro gel dissolution time of 22 days [13]. In vitro bupivacaine release from SBG004 continued over more than 240 h; the fraction of bupivacaine released was 2.8 ± 0.2% at 1 h, 23.0 ± 2.0% at 48 h, 54.4 ± 4.0% at 144 h, and 83.0 ± 3.5% at 240 h (mean ± s.d.).

### 2.2. Pharmacokinetics Following Periarticular and Intraarticular Injection

**Figure 1:**
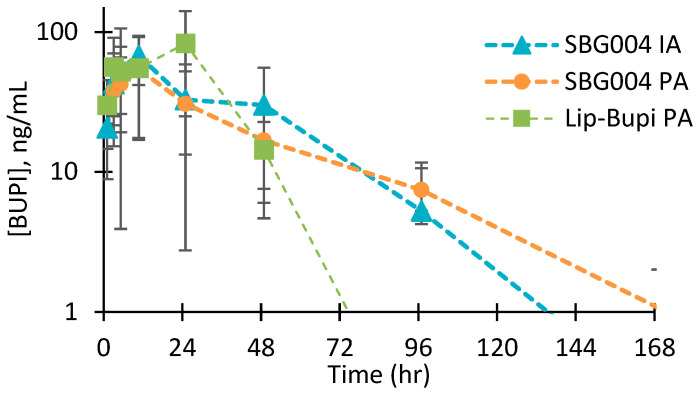
Bupivacaine concentration in serum following dosing of SBG004 PA, SBG004 IA, and undiluted Lip-Bupi PA (n = 4, mean ± s.d.).

**Figure 2:**
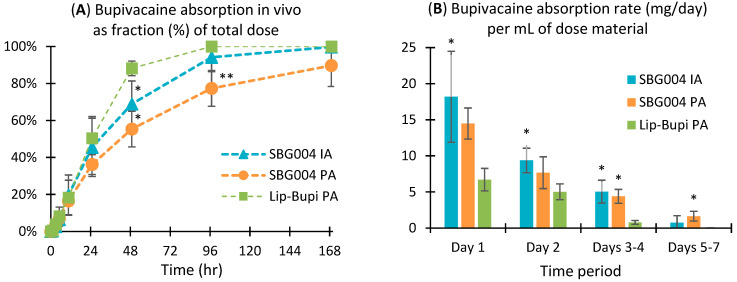
(**A**) Fraction of bupivacaine absorbed following dosing with SBG004 and Lip-Bupi. (**B**) Bupivacaine absorption rate per mL of dose material during selected time periods. Both represent n = 4, mean ± s.d. * = significant vs. Lip-Bupi PA, ** = significant vs. SBG004 IA and Lip-Bupi PA (*p* < 0.05, Tukey’s HSD test).

Bupivacaine exposure was measured in all four rabbits per group through 168 h post-dose for rabbits receiving SBG004 PA, 96 h for SBG004 IA, and 48 h for Lip-Bupi PA (Figure 1). Pharmacokinetic parameters are shown in Table 1. SBG004 PA was associated with the greatest half-life (49.6 ± 19.4 h) and lowest C_max_ (60.7 ± 29.7 ng/mL). Lip-Bupi PA had the shortest half-life (6.9 ± 1.7 h) and highest C_max_ (97.6 ± 50.6 ng/mL). The fraction of bupivacaine absorbed into the circulation through 49 h was 55 ± 10% for SBG004 PA, 69 ± 12% for SBG004 IA, and 88 ± 4% for Lip-Bupi PA (Figure 2A). The average rate of bupivacaine absorption per mL of dose material was continuously greater for both of the SBG004 groups compared to Lip-Bupi PA (Figure 2B).

### 2.3. Analgesic Efficacy in Rabbits

There was substantial agreement [15] among the three observers in their rating of videos as indicating either weight-bearing, lack of weight-bearing, or no scorable steps (Fleiss’ kappa = 0.62) for the full set of 419 videos before committee review. Among the 378 videos for which individual observer scores were used in the final analysis (i.e., excluding those reviewed by committee), Fleiss’ kappa was 0.68 and there was unanimous agreement on 278 videos (73.5%).

**Figure 3:**
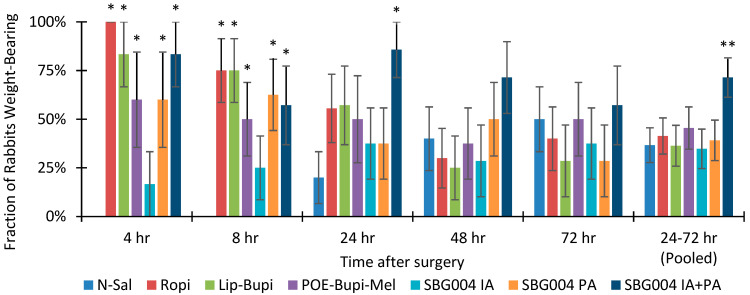
Fraction of rabbits weight-bearing during spontaneous ambulation following knee surgery (data combined for both sexes, mean ± SEM, n = 5–10). * = significant vs. N-Sal; ** = significant vs. all other groups except POE-Bupi-Mel (*p* < 0.05, Fisher’s exact test).

Weight-bearing data are shown in Figure 3 and reflected in tabular form in Table S1. The proportion of weight-bearing rabbits was greater in all active groups except SBG004 IA compared to normal saline (N-Sal) at 4 h and 8 h postoperatively. No rabbits in the N-Sal group were weight-bearing at 4 h or 8 h postoperatively (0/8 and 0/10, respectively). Only the group receiving SBG004 IA + PA had significantly higher weight-bearing compared to N-Sal at 24 h (6/7 versus 2/10, *p* = 0.015). There were no significant differences in the proportion of weight-bearing rabbits at 48, 72, or 96 h postoperatively. SBG004 IA + PA was the only group in which over half of the rabbits were weight-bearing at 48 h and 72 h (5/7 and 4/7, respectively). Considering all data from 24 to 72 h, rabbits receiving SBG004 IA + PA were weight-bearing in 71% (15/21) of videos, whereas the proportion of weight-bearing rabbits in all other groups was in the range of 35–45% (*p* < 0.05 vs. all other groups except POE-Bupi-Mel).

## 3. Discussion

In this work, we studied the pharmacokinetics of SBG004 and Lip-Bupi using routes of administration relevant to knee surgery. The data indicate that the rank order for bupivacaine release, slowest to fastest, is SBG004 PA, SBG004 IA, and then Lip-Bupi PA. The pharmacokinetic profiles in this work are in reasonable agreement with prior reports of pharmacokinetics following subcutaneous dosing for both SBG004 [13] and Lip-Bupi [16]. SBG004 IA may have released bupivacaine more rapidly than SBG004 PA due to exposure to external forces within the joint; however, release was still steady without high initial burst. Systemic absorption data indicate that the rate of bupivacaine release from SBG004 PA or SBG004 IA would be expected to be continuously greater than from an equivalent volume of Lip-Bupi PA. In other words, the slower drug release profile from SBG004 is more than offset by the higher drug loading of SBG004 compared to Lip-Bupi. This suggests that SBG004 sustains greater local bupivacaine concentration compared to Lip-Bupi throughout the course of moderate to severe postoperative pain in applications where the dose is limited by volume rather than risk of systemic toxicity.

SBG004 contains two phases: a gel phase containing water and PNDJ and a particulate phase of bupivacaine and PEG300. Slow bupivacaine release from SBG004 likely arises from two processes. First, bupivacaine must dissolve from the particulate phase into the aqueous PNDJ-containing phase. The initial bupivacaine concentration in the particulate phase (20 wt%) is approximately 1000-fold greater than the solubility of bupivacaine in the aqueous phase at physiological pH (200 ppm) [17]. Thus, the particulate phase functions to maintain the concentration of soluble drug within the gel phase near solubility. Second, diffusion of soluble bupivacaine within the PNDJ gel phase is slowed due to the effective high viscosity of the PNDJ in its gelled state; this mechanism on its own has been utilized for extended release of water-soluble small molecules from similar gels [18,19]. Release from Lip-Bupi is thought to be mediated by multiple mechanisms, including destabilization or erosion of the multivesicular liposome structure, lipid rearrangement, and permeation of lipid membranes [20,21,22].

We also studied analgesia provided by three extended-release local anesthetics and periarticular local anesthetic cocktail following knee surgery in rabbits. The pharmacokinetic data establish that bupivacaine release continues for over 96 h from SBG004 and for over 48 h from Lip-Bupi in the same species and routes as in the analgesia study. Similarly, pharmacokinetic data from a study of POE-Bupi-Mel in human patients undergoing TKA suggest release continues for over 72 h [6]. The purpose of the analgesia experiment in this work was to determine whether each treatment’s combination of release profile, dose, and spatial distribution provided sufficient analgesia to allow spontaneous weight-bearing. Significantly higher weight-bearing rates versus N-Sal were observed for rabbits receiving SBG004 IA + PA through 24 h postoperatively and for rabbits receiving Ropi, Lip-Bupi, POE-Bupi-Mel, and SBG004 PA through 8 h postoperatively. SBG004 IA was ineffective. No group achieved significance compared to N-Sal at 48 or 72 h postoperatively, which would have required a weight-bearing rate of 100%. Considering pooled data from 24 to 72 h, the weight-bearing rate for rabbits receiving SBG004 IA + PA was significantly higher than all other groups except POE-Bupi-Mel. No other active group was significantly superior to N-Sal during this period.

A limitation of this work was its reliance on weight-bearing for assessment of postoperative pain. We initially assessed pain-related behaviors using a Rabbit Pain Behavior Scale (RPBS) which was developed in research rabbits undergoing painful procedures, including partial radial ostectomy [23,24]. However, even when criteria of the scale were modified to distinguish among the range of behaviors observed in this study, there was no difference between the negative control (N-Sal at 4 and 8 h) and positive control (Ropi cocktail at 4 and 8 h) groups for the total score or most subcategories, including posture, activity, interaction/appetite, facial expression, licking the site, and miscellaneous behaviors such as twitching and wincing (Supplementary Materials, Table S2, Figures S2–S11). Among the outcomes considered in the RPBS, we found that weight-bearing was the best indicator of pain. For example, at 4 h when Ropi can be considered a positive control, 10/10 rabbits receiving Ropi were scored as weight-bearing versus 0/8 rabbits receiving N-Sal. Weight-bearing is also specific to use of the joint, whereas other potentially pain-related behaviors are confounded by recovery from sedation, skin irritation, or acclimation to the cage environment.

A second limitation of this work was that assessment of weight-bearing behavior was subjective and based on video review. Not all steps could be included due to an obscured view of the hindlimb. We mitigated potential bias by having multiple observers score each video in a random order while blinded to the treatment group and time point. Committee review was performed for videos in which agreement between observers was poor, representing less than 10% of the videos in the study. The study could have been improved by including objective measurement of weight-bearing in a given step.

A third limitation was that the study was underpowered to detect differences of up to 50% in weight-bearing as significant at any individual time point. Additionally, the capability for the model to detect analgesia declined beginning at 24 h postoperatively because the weight-bearing rate in the N-Sal group increased, as expected. At 48 h and 72 h, a 100% weight-bearing rate in a group was required to reach significance compared to N-Sal (100% would not reach significance at 96 h). No treatment in this study resulted in the absence of pain necessary to meet this criterion. When considering all data from 24 to 72 h (n = 21–30), reaching statistical significance would require about a 30% difference in weight-bearing rate. We believe this would indicate a clinically meaningful reduction in pain. The use of pooled data over the period from 24 to 72 h is analogous to outcomes used in clinical studies of ERLAs [6,25,26,27,28,29], including the AUC of the pain intensity score and total opioid consumption.

A fourth limitation was that data from 25 videos (6.0%) were excluded from analysis due to the rabbits not walking. The greatest incidence occurred at 4 h postoperatively, which was likely due to recovery from anesthesia. Lack of walking at later time points was predominantly attributed to resting. Almost all of the rabbits in the study rested for a majority of the recorded time. A typical behavior pattern consisted of first eating the lettuce and yogurt, then exploring the cage for 2–10 min, and then resting for the remaining time.

A fifth limitation was that the pharmacokinetics of three extended-release test treatments (Lip-Bupi cocktail, SBG004 IA + PA, and POE-Bupi-Mel) were not measured in the pharmacokinetic study. In each case, the duration and extent of systemic exposure can be estimated on the basis of available data involving PA or IA dosing. Exposure from Lip-Bupi cocktail can be estimated based on the sum of exposure from the Lip-Bupi component and the plain bupivacaine component of the cocktail. Exposure of SBG004 IA + PA (2 mL IA and 3 mL PA) can be estimated as double the exposure from 1 mL SBG004 IA plus triple the exposure from 1 mL SBG004 PA. This method would probably overestimate systemic exposure as release should decline somewhat with the decreased surface-area-to-volume ratio of the gel that SBG004 forms in situ. POE-Bupi-Mel was not studied in this work, but pharmacokinetic data from human TKA indicate continuous systemic exposure for over 72 h [6].

The range of behaviors observed in the analgesia study varied within the broad categories of weight-bearing or non-weight-bearing. Among weight-bearing rabbits, a relatively small number exhibited gait that was nearly normal with little to no favoring of the operated limb and simultaneous push off with both hindpaws; this was only common in the Ropi group at 4 h postoperatively. More often, weight-bearing on the right hindpaw appeared light, including an early lift-off with the right hindpaw. During non-weight-bearing steps, rabbits would lift or drag the right hindpaw but would still land on the paw and touch or drag the foot on the cage floor during the step. A more extreme display of pain was suspending and protecting the limb throughout the step. The only subset for which this was the most common behavior was the negative control group at 4 h postoperatively.

While the 0–4 scale was used to screen for interobserver discrepancies, we chose to classify rabbits as weight-bearing or not for two reasons. First, we judged that the most clinically important difference in the observed behaviors was between weight-bearing and non-weight-bearing, which indicates whether a rabbit either did or did not tolerate its level of pain. Second, we found that distinguishing between full, moderate, and light weight-bearing (scores of 0, 1, or 2) was difficult to interpret, relying on subtle indications like relative timing of the hindpaws lifting off. Assessment of presence or absence of weight-bearing was more reliable as it relied on ground reaction leading to dorsiflexion of the toes and the orientation of the foot during the step.

Although SBG004 IA + PA did not support 100% weight-bearing at any time point, it was associated with prolonged analgesia, including improved weight-bearing rate over the period of 24–72 h compared not only to N-Sal but also active groups, including Lip-Bupi and Ropi. This is consistent with its release duration of over 96 h, higher bupivacaine loading, high dose volume, and thorough distribution around the joint.

A dose of 1 mL SBG004 was not effective by PA injection after 8 h or by IA injection at any time point. We attribute this to slow release for both routes and insufficient spatial distribution of the dose, failing to sustain sufficient bupivacaine concentrations throughout local tissues. Using absorption data calculated from the pharmacokinetic study results as a measure of in vivo drug release, we estimate that bupivacaine was released from 1 mL SBG004 at a rate of about 0.58–0.73 mg/h over the first 24 h, whereas the ropivacaine group contained 8.68 mg ropivacaine which was immediately available. An increased dose volume of 5.2 mL was evaluated to increase the drug release rate, overcome washout from the local tissue, and improve spatial coverage, which may be critical given the anatomic complexity of sensory innervation of the knee.

SBG004 IA 1 mL was ineffective at all time points despite higher release than SBG004 PA 1 mL as indicated by the pharmacokinetic data. This is similar to our previous results in a porcine skin and muscle incision model, in which we found that SBG004 was more effective when given by peri-incisional subcutaneous injection compared to wound filling [13]. These findings mutually indicate that blocking surgical pain requires diffusion substantially into all tissues where afferent nerve fibers are located, which is not achievable from within the surgical wound space. Consistent with this, in vivo [30] and ex vivo [31] studies have found that soluble molecules penetrate poorly (no more than about 1 mm) into intact muscle. Clinical technique reflects this understanding as well; for example, in abdominal surgery, surgeons infiltrate plain LA solution at a depth of 0.5–1 cm rather than filling the wound [32]. The lack of effect of SBG004 IA suggests that most of the effect of SBG004 IA + PA was due to the PA fraction of the dose.

Lip-Bupi and POE-Bupi-Mel yielded similar outcomes to Ropi in this study. Weight-bearing rates in these groups were 30–37% greater than N-Sal at 24 h, suggesting a potential analgesic effect that did not meet the threshold for significance. There was no indication of analgesia in these groups at 48 h or later. Although ropivacaine alone is known to have a shorter duration of no more than 6 h [33], it is possible that the inclusion of ketorolac and epinephrine in the cocktail extended its effect to 8 h and possibly 24 h. A notable finding for Lip-Bupi was that 38 ± 8% of bupivacaine absorption occurred in the period from 24–48 h, whereas its analgesic efficacy both in this study and in clinical studies [34] is generally more attenuated. The lack of efficacy beyond 24 h may be attributable to washout of liposomes from local tissue, resulting in release which contributes to systemic absorption but not local analgesic efficacy. Efficacy of POE-Bupi-Mel was likely hindered by the relatively low maximum feasible dose in the model (about 0.5 mL). While this is nearly proportional to the dose of POE-Bupi-Mel indicated for TKA in humans, increased surface area to volume could have contributed to faster release. Another likely contributing factor is limited penetration into tissue due to dosing of POE-Bupi-Mel within the surgical wound space.

To our knowledge, this is the first experiment comparing multiple ERLAs in a nonrodent orthopaedic model. Using a similar surgical model in rats, Buvanendran et al. reported that the difference in rearing activity between rats undergoing the procedure without analgesics and rats undergoing sham surgery declined over 3–4 days postoperatively as the rats adapted to their environment [35]. We observed a similar time course in this work. As a result, the capability of the model to determine analgesia declined as responses in the N-Sal group improved beginning at 24 h postoperatively. Buvananendran et al. subsequently reported that local infiltration with a cocktail of bupivacaine, ketorolac, and dexamethasone reduced rearing activity by about 30–50% at 2 h and 1 day postoperatively [36]. In this work, we observed a greater difference in the incidence of weight-bearing between rabbits receiving plain local anesthetic infiltration at 4 h and 8 h compared to those receiving N-Sal, with no weight-bearing in the N-Sal group and near universal weight-bearing in the Ropi group. Thus, weight-bearing in the rabbit may be a clearer indicator of analgesia than rearing in the rat, which can occur without bearing weight on the operated limb.

The most commonly used model for evaluation of ERLAs is a porcine soft tissue incision model with assessment of mechanical allodynia initially published by Castel et al. [37]. Versions of this model have been used to evaluate the duration of analgesia provided by plain local anesthetics [13,38], Lip-Bupi [13,38,39], POE-Bupi-Mel [13,39,40], and SBG004 [13]. The durations of analgesia we measured in this work are similar to reported results in the porcine incision model for plain local anesthetics and Lip-Bupi. For POE-Bupi-Mel, our finding of analgesia lasting 8–24 h in knee surgery is slightly longer than our previous finding of 4 h following skin and muscle incision [13] and similar to Yang et al. [40], who reported an increased withdrawal threshold through 24 h. However, Ottoboni et al. reported a longer duration of 72 h [39]. Prolonged analgesia observed in that study may be attributed to POE-Bupi-Mel being injected subcutaneously in the wound margins, which is not consistent with its instructions for clinical use [41] and would overcome the aforementioned limitation of poor diffusion from the surgical wound space.

The model used in this work may understate the potential for clinically significant analgesia. For example, POE-Bupi-Mel did not lead to increased weight-bearing versus N-Sal at 24 h or 48 h in this study, yet it was effective in a Phase 2b clinical study in patients undergoing TKA, reducing average pain scores by 1–2 points on a 10-point scale versus N-Sal for up to 60 h postoperatively [6].

The in situ gelling property of SBG004 appears to be advantageous for local anesthetic delivery by combining injectability and retention at the injection site. Injectability appears to be required for direct drug delivery throughout a sufficient zone of local tissue to cause analgesia in this model and our previous soft tissue model [13]. A secondary benefit of injection is that the dosing technique is similar to clinical use of plain LAs. Retention of the dose at the injection site supports releasing the local anesthetic at the injection site for the duration of drug release, whereas particulate or soluble drug delivery systems that float in fluid (liposomes, microparticles, prodrugs, etc.) are prone to dilution over more than 24 h. These spatial considerations suggest that formulations which either release local anesthetic within the surgical wound space itself or particulate systems prone to dilution may be inherently limited regardless of release kinetics or drug loading.

## 4. Materials and Methods

### 4.1. Materials for PNDJ Synthesis and SBG004 Preparation

All chemicals were reagent grade and purchased from Sigma-Aldrich (St. Louis, MO, USA) unless otherwise stated. Heptane and methyl tert-butyl ether (MTBE) were purchased from Oakwood Chemical (Estill, SC, USA). Dimethyl butyrolactone acrylamide (DBLAAm) was obtained from Enamine (Monmouth Junction, NJ, USA). Jeffamine M-1000 was obtained from Huntsman (The Woodlands, TX, USA). Bupivacaine free base was obtained from Cayman Chemical (Ann Arbor, MI, USA). Polyethylene glycol 300 (PEG300, MW 300 g/mol) was obtained from Tex Lab Supply (Lubbock, TX, USA).

### 4.2. PNDJ Synthesis and Characterization

The PNDJ from this work was from the same lot as our previous report [13]. PNDJ was synthesized by free radical polymerization of *N*-isopropylacrylamide, DBLAAm, and Jeffamine M-1000 acrylamide. The three monomers (molar ratio 77.77: 20.50: 1.73) were added to a total concentration of 10 w/v% to a reaction flask containing equal volumes tetrahydrofuran and dioxane pre-heated to 65 °C. Polymerization proceeded following addition of 0.007 mol azobisisobutyronitrile per mol of total monomer. After 18 h, the reaction solution was precipitated in eight-fold volume excess of heptane, filtered, and vacuum-dried. The resulting crude polymer was then redissolved to about 25 w/v% in acetone and precipitated in MTBE at the same volume as heptane in the first precipitation step (about 27-fold volume excess), followed by filtration, rinsing with excess MTBE, filtration, and vacuum-drying. The processes of dissolution in acetone and precipitation, rinsing, and filtration with MTBE were repeated once more to further remove residual solvents and free monomers. PNDJ was obtained as a white powder and stored at −20 °C until use. Repeat unit content was determined by ^1^H NMR (400 MHz, Varian, Palo Alto, CA, USA) in CD_3_OD. Molecular weight was determined by gel permeation chromatography including a Styragel HR4 THF column (Waters, Milford, MA, USA), multi-angle light scattering detector (MiniDawn, Wyatt Technology, Santa Barbara, CA, USA), and refractive index detector (RID-10A, Shimazdu, Columbia, MD, USA).

### 4.3. SBG004 Preparation and Characterization

PNDJ was dissolved at 33 wt% in 20 mM acetic acid–sodium acetate (pH 4.0), sterilized by filtration, aseptically loaded into syringes, and stored at 2–8 °C. Separately, bupivacaine was combined with PEG300 in a 1:4 ratio by mass, heated to 100 °C to form a transparent colorless solution, and aseptically loaded into separate syringes which were stored at 2–8 °C. SBG004 was prepared by syringe-mixing 33 wt% PNDJ solution in a 4:1 ratio by mass with bupivacaine/PEG300, resulting in 40 mg bupivacaine per gram of SBG004.

Viscosity, gelation temperature, and in vitro gel dissolution time of SBG004 were measured in previous experiments for the batch used in this work [13]. Viscosity at 5 °C and gelation temperature were measured by rheometry (MCR-101, Anton Paar USA, Ashland, VA, USA). Viscosity was measured at 1/sec shear rate. Gelation temperature was the temperature at which complex modulus exceeded 50 Pa at 0.5% strain under oscillation applied at 1 Hz while heating at 2 °C/min beginning at 5 °C. In vitro gel dissolution time was determined using 300 mg gels in 8 mL excess PBS (pH 7.4) at 37 °C with daily buffer exchange. The dissolution time was the time at which the gel was not retained as a single cohesive mass when the vial was inverted.

In vitro bupivacaine release was measured using a compendial rotating basket dissolution apparatus (2100C, Distek, North Brunswick, NJ, USA). SBG004 samples (n = 6) of about 1 g each were dispensed into the larger side of Size 000 pullulan capsules (PureCaps, Canton, OH, USA) with the actual weight recorded. The half-capsules full of liquid SBG004 were then rapidly transferred to the baskets, inside which they rested at an angle but did not fall over or spill. Dispensing of all samples required approximately five minutes. After all samples were dispensed, baskets were attached to the apparatus and lowered into the release medium. All vessels were pre-warmed to 37 °C and contained 750 mL of freshly de-aerated PBS (pH 7.4). Baskets were rotated at 50 rpm. Samples (1 mL) were collected without buffer replacement at 1, 24, 48, 72, 144, 240, and 336 h. Samples were analyzed for bupivacaine concentration by HPLC-UV (1100 series, Agilent Technologies, Santa Clara, CA, USA). The method included a flow rate of 1 mL/min, sample injection volume of 15 µL, and a mobile phase consisting of various ratios of 0.01% trifluoroacetic acid in water (A) and acetonitrile (B). The composition of the mobile phase (%B) was a linear increase from 15 to 45% from t = 0–6 min, 45–90% from t = 6–7 min, constant at 90% from t = 7–8 min, and constant at 15% from t = 8–11 min. The wavelength for detection was 263 nm. The retention time of bupivacaine was approximately 6.7 min. The measured bupivacaine concentration in the medium was normalized to the measured mass of each gel sample assuming a drug loading of 40 mg bupivacaine per 1 g gel.

### 4.4. Preparation of Other Dose Materials

Ropivacaine-based cocktail (Ropi) (ropivacaine 1.67 mg/mL and ketorolac 0.2 mg/mL (both Fresenius Kabi, Lake Zurich, IL, USA) and epinephrine 0.83 µg/mL (Par Pharmaceuticals, Chestnut Ridge, NY, USA) was prepared aseptically using solutions from pharmacy vials and normal saline from 10 mL prefilled syringes (BD, Franklin Lakes, NJ, USA). Liposomal bupivacaine-based cocktail (Lip-Bupi) (2.22 mg/mL liposomal bupivacaine, 1.04 mg/mL bupivacaine, 0.25 mg/mL ketorolac, 1.04 µg/mL epinephrine) was prepared by first combining bupivacaine HCl, ketorolac tromethamine, epinephrine, and normal saline aseptically within three days prior to dosing and then mixing immediately prior to dosing with liposomal bupivacaine suspension (Exparel, Pacira Biosciences, Parsippany, NJ, USA). Exparel is a proprietary multivesicular liposome suspension that contains 13.3 mg/mL bupivacaine [42]. A cocktail containing liposomal bupivacaine and free bupivacaine was used to replicate common clinical practice in TKA, including work presently referenced by the manufacturer for administration in TKA [43]. The compositions of each cocktail were adapted from those used by Amundson et al. for periarticular infiltration in human TKA [12]. Bupivacaine–meloxicam polyorthoester (POE-Bupi-Mel) solution (Zynrelef, Heron Therapeutics, San Diego, CA, USA) was dispensed according to the manufacturer’s instructions. Normal saline (N-Sal) doses were given from 10 mL prefilled syringes (BD).

### 4.5. Pharmacokinetics Following Periarticular and Intraarticular Injection

New Zealand White rabbits (female, 2.5–3 kg, n = 4) were randomized to receive either (i) SBG004 14.5 mg/kg PA, (ii) SBG004 14.5 mg/kg IA, or (iii) Lip-Bupi 14.5 mg/kg PA. In this study, Lip-Bupi suspension was used without dilution (13.3 mg/mL), not as a cocktail. Blood was collected via a catheter from the marginal ear vein for measurement of serum bupivacaine concentration at approximately 1, 3, 5, 12, 24, 48, 96, 168, and 336 h post-dose. Actual dosing and blood collection times were recorded and used in calculation of pharmacokinetic (PK) parameters for each rabbit. Bupivacaine concentration in each sample was measured using LC-MS/MS by Texas A&M Veterinary Medical Diagnostic Laboratory (College Station, TX, USA) [44]. Pharmacokinetic parameters were calculated using PKSolver in Microsoft Excel, assuming a noncompartmental model with extravascular input [45]. Differences in pharmacokinetic parameters were evaluated by ANOVA with Tukey’s HSD post hoc test to evaluate differences between each pair of groups (α = 0.05).

The fraction of absorbed bupivacaine for each group as a function of time was calculated as *AUC*(*0*−)/*AUC*(*0* − *∞*), and the rate of bupivacaine absorption per day per mL of dose material was calculated for each interval (Day 1, Day 2, Days 3–4, and Days 5–7) by multiplying the fraction of absorbed bupivacaine during the interval by the bupivacaine loading of the test material and dividing by the duration of the interval. The former quantity compares the drug release profile for an equal dose whereas the latter accounts for the higher drug loading of SBG004 compared to Lip-Bupi. Data for each interval were analyzed by ANOVA with Tukey’s HSD post hoc test (α = 0.05).

### 4.6. Surgical Procedure & Recovery

The surgical procedure used in this work was based on that of Buvanendran et al., in which holes were drilled in the distal femur and proximal tibia in rats [35]. The procedure was reported to produce pain for up to three days postoperatively as measured by spontaneous exploratory behavior (rearing count) and response to joint squeezing. Pain in this model was mitigated using local infiltration analgesia [36]. We adapted the surgical procedure to a rabbit model because the larger size allows for more consistent dosing of viscous test substances and more similar surface area to volume ratio compared to humans, which could affect drug release kinetics from SBG004.

**Figure 4:**
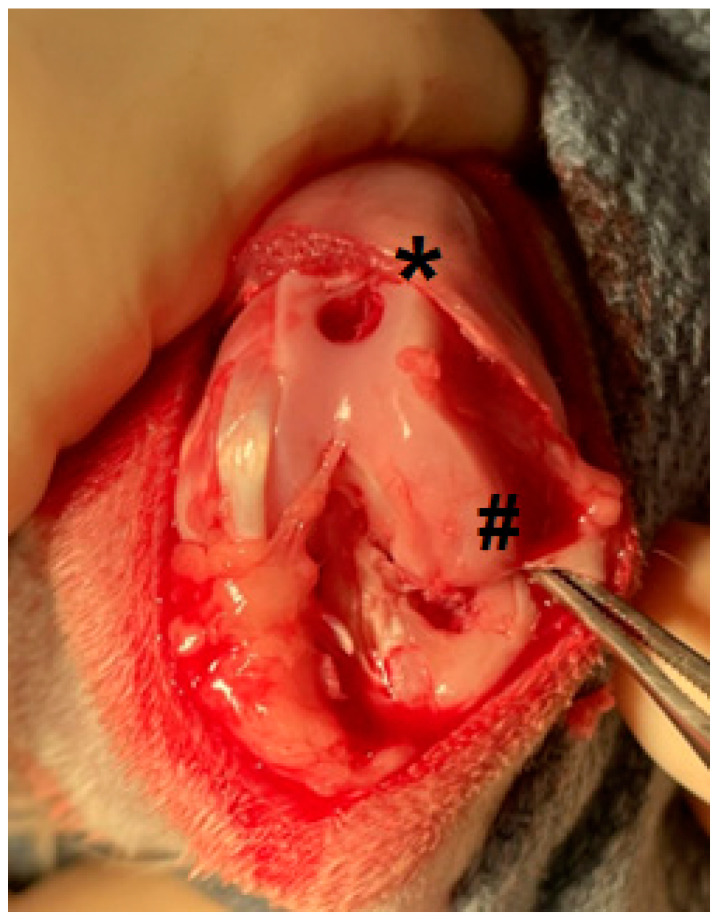
Image of the joint before test article dosing in the rabbit knee surgery model, showing defects created in the femoral groove (*) and tibial plateau surfaces (**#**).

All procedures were performed in the vivarium at St. Joseph’s Hospital and Medical Center (Phoenix, AZ, USA) according to a protocol approved by the Institutional Animal Care and Use Committee and in compliance with all relevant legislation. New Zealand White rabbits (male and female, 2.5–3 kg) were singly housed with ad libitum food and water and a toy for enrichment. Hay was given daily. Rabbits underwent surgery on the right hindlimb. Rabbits were sedated using ketamine (15–35 mg/kg IM) and medetomidine (0.25–0.5 mg/kg IM) and anesthetized with 1–4% isoflurane in oxygen. A 3–4 cm anterior skin incision was made followed by a medial parapatellar arthrotomy. The patella was dislocated and then the knee was flexed beyond 90°. Using a handheld chuck, 3 mm diameter holes were drilled into the articular cartilage and subchondral bone of the femoral groove and medial tibial plateau, not penetrating the medullary canal. The distal medial femoral condyle articular cartilage was abraded by the drill bit while creating the hole in the tibia (Figure 4). The rabbit was then randomly assigned to receive a specific treatment. The route of administration for test materials was intraarticular (IA), periarticular (PA), both intraarticular and periarticular (IA + PA), or coating wound surfaces (WSs) as explained in the study design below. The surgical site was closed in layers. Rabbits received 1 mg/kg meloxicam SC intra-operatively for perioperative analgesia [46]. Atipamezole (0.35 mg/kg SC) was given postoperatively for reversal of sedation. The protocol allowed for rescue analgesia (buprenorphine 0.01–0.05 mg/kg SC) for any rabbit determined by veterinary staff to be experiencing more than momentary severe pain or discomfort after 8 h postoperatively. No rabbits were determined to meet this criterion except for one rabbit euthanized due to presumed sepsis secondary to GI stasis as described in Section 4.8.

### 4.7. Study Design

The study was completed in two phases; the study methods and process used for data analysis are summarized in Figure S1. In the first phase, 48 rabbits (n = 4/sex/group) were randomized to receive one of six treatments: (i) SBG004, 1 mL PA; (ii) SBG004, 1 mL IA; (iii) Lip-Bupi, 5.2 mL PA; (iv) POE-Bupi-Mel, approx. 0.5 mL WS; (v) Ropi, 5.2 mL PA; or (vi) N-Sal 5.2 mL PA (Table 2). Lip-Bupi and POE-Bupi-Mel are active comparator groups using commercially available extended-release local anesthetics. Ropi is an active comparator group that was considered to be a positive control at 4 h postoperatively. N-Sal is a negative control.

SBG004 IA was injected via 18G needle after closure. For all PA dosing of all agents (SBG004 PA, Lip-Bupi, Ropi, and N-Sal), injection was into the periarticular soft tissues using a 20G × 1” needle (18G × 1” for SBG004) just superficial to the medial, lateral, and posterior capsule and the quadriceps and patellar tendon regions such that the infiltrated agent was distributed continuously in the soft tissues surrounding the knee similar to the technique used following human TKA. Doses for Lip-Bupi and Ropi were scaled to body weight compared to expected or published dosing for TKA in a 70 kg human [12]. Saline was given at the same volume. The dose of POE-Bupi-Mel was the maximum feasible volume administered by coating the joint surfaces according to the manufacturer’s instructions for TKA.

In the second phase, 12 rabbits were randomized to receive either SBG004 5.2 mL IA + PA (n = 4/sex), Ropi 5.2 mL PA (n = 1/sex), or N-Sal 5.2 mL PA (n = 1/sex). In the SBG004 IA + PA group, approximately 3 mL was infiltrated PA as described above and 1.5–2 mL (max possible) was injected IA after closure.

### 4.8. Data Collection and Scoring

Spontaneous behavior was recorded for 20 min periods in each rabbit’s home cage using a camera mounted approximately 2 ft from the front of the cage between 24 and 72 h before surgery (pre-op), and at 4, 8, 24, 48, 72, and 96 h postoperatively. Before each recording period, rabbits received a piece of lettuce covered with a thin layer of fruit-flavored yogurt on a plate and an additional enrichment toy to motivate activity. Food trays mounted on the cage door were temporarily removed during recording to avoid obstructing the view of the rabbit. The technician left the room before recording began.

Video files for each rabbit and time point were de-identified and renamed in random order for scoring. Three observers scored each video by evaluating weight-bearing on the right (operated) hindlimb on each step that met the following criteria: (i) all four paws and the body of the rabbit moved forward by more than about 10 cm in a continuous motion; (ii) both hind paws could be seen during pushing off on the cage floor; (iii) the rabbit did not change direction during the step by more than 90°. Weight-bearing could not be assessed reliably for small “shuffle” steps where only the hindlimbs moved forward while the forepaws remained stationary. Observers made independent determinations of whether a given step met the criteria for being scored. The assigned score for each step was an integer from 0 to 4, where 0 = normal weight-bearing; 1 = partial weight-bearing with moderate push-off, minimally favoring the right hindlimb; 2 = partial weight-bearing with light push-off, clearly favoring the right hindlimb; 3 = no weight-bearing but touching or dragging the toes during the step; and 4 = no weight-bearing while holding the right hind limb up during the step.

A total of 419 videos were analyzed out of 420 possible. One time point from one rabbit (POE-Bupi-Mel group, 96 h) was excluded due to the rabbit being euthanized prior to the time point; this rabbit began to have diarrhea and lethargy which was attributed to sepsis from GI stasis. Videos judged by at least two of the three observers to have no scored steps were analyzed as showing no walking.

A committee review process was used to resolve major interobserver differences prior to data analysis. Following independent scoring by the three observers, videos were selected for committee review if either the difference in mean step score between any two observers was 2.0 points or greater (38 videos, 9.1%) or if one of the three observers scored no steps (3 videos, 0.7%). During committee review, all steps previously scored by any observer were reviewed. The three observers discussed and voted on a consensus score for each step or agreed to not count the step. In the case of continued disagreement, a two-thirds vote was taken to assign a score or not count the step. The committee’s scores for these videos were then used in place of the individual observer scores. In summary, the data following committee review consisted of individual step scores for 378 rabbit/time point combinations by three observers and the individual step scores for the remaining 41 rabbit/time point combinations as determined by committee review.

Weight-bearing status was then determined as a binary outcome for each rabbit/time point combination with scored steps (385 videos, 91.9%). A rabbit/time point combination was classified as weight-bearing if 50% or more of the steps were scored as 0, 1, or 2 on the 0–4 scale by at least two of the three observers or by committee scores. Thirty-four videos (8.1%) were determined to have no scorable steps, including 25 videos with no walking and 9 videos showing walking that did not meet the criteria for scoring a step. These rabbit/time point combinations were excluded from analysis because weight-bearing could not be determined. Fourteen such videos were recorded 4 h postoperatively; there were no more than six at any other time point. There were no significant differences in the frequency of no scored steps between groups at any time point (Table S3).

### 4.9. Data Analysis

Statistical analysis was performed using Real Statistics Resource Pack (https://real-statistics.com/free-download/, accessed on 14 April 2024) in Microsoft Excel Version 2405 [47]. Interobserver agreement on weight-bearing status (weight-bearing, non-weight-bearing, or no scored steps) was assessed by Fleiss’ kappa. Data from both sexes were combined for analysis; the study was not powered to detect differences between sexes which would not be expected based on clinical use of bupivacaine. Differences in weight-bearing status for each active group versus N-Sal and for SBG004 IA + PA versus other groups at each time point were evaluated by Fisher’s exact test. Pooled data from the period of 24–72 h were also analyzed as this time is particularly important for supporting discharge to self-care. The significance level α was 0.05 for all tests.

## 5. Conclusions

SBG004 resulted in prolonged analgesia compared to the clinically available treatments ropivacaine-based cocktail, liposomal bupivacaine-based cocktail, and polyorthoester bupivacaine–meloxicam in rabbit knee surgery. SBG004 is differentiated by its in situ gelling property which enables injection and retention in tissue throughout the duration of moderate to severe postoperative pain. The results support further development of SBG004, including definitive nonclinical toxicology studies prior to potential evaluation in clinical studies. In future work, studies comparing the distribution of local anesthetics in tissue provided by extended-release formulations should be considered.

## Figures and Tables

**Table 1:** Selected pharmacokinetic parameters for released bupivacaine.

Parameter	Unit	SBG004 IA, 14.5 mg/kg	SBG004 PA, 14.5 mg/kg	Lip-Bupi PA, 14.5 mg/kg
t_1/2_	h	20.1 ± 5.0	49.6 ± 19.4 **	6.9 ± 1.7
t_max_	h	9.3 ± 2.8	7.4 ± 3.9	19.6 ± 11.0
C_max_	ng/mL	86.8 ± 37.6	60.7 ± 29.7	97.6 ± 50.6
AUC (0–336 h)	ng/mL × h	2980 ± 1500	2630 ± 1520	2990 ± 1720
C_max_/Dose	(ng/mL)/(mg/kg)	5.96 ± 2.58	4.17 ± 2.04	6.71 ± 3.48

** significant vs. SBG004 IA and Lip-Bupi PA (*p* < 0.05, Tukey’s HSD test).

**Table 2:** Analgesic efficacy study design in a rabbit knee surgery model.

				Number of Rabbits		
Agent	Dose	Route	Purpose	Phase 1	Phase 2	Total
N-Sal	5.2 mL	PA	Negative Control	8	2	10
Ropi	5.2 mL	PA	Active Control	8	2	10
Lip-Bupi	5.2 mL	PA	Active Control	8	-	8
POE-Bupi-Mel	Max. feasible	WS	Active Control	8	-	8
SBG004	1 mL	PA	Experimental	8	-	8
SBG004	1 mL	IA	Experimental	8	-	8
SBG004	5.2 mL	IA + PA	Experimental	-	8	8

N-Sal = saline, Ropi = ropivacaine cocktail, Lip-Bupi = liposomal bupivacaine cocktail, POE-Bupi-Mel = bupivacaine–meloxicam polyorthoester PA = periarticular injection; IA = intraarticular injection; WS = surgical wound surface coating.

## Data Availability

The raw data supporting the conclusions of this article will be made available by the authors on request.

## Supplementary Materials

The following supporting information can be downloaded at: https://www.mdpi.com/article/10.3390/ph17070879/s1, Table S1. Fraction of rabbits that were weight-bearing in rabbit knee surgery study; Table S2. Modified rabbit pain behavior scale and purpose of modifications made; Table S3. Frequency of no scored steps in rabbit knee surgery study; Figure S1. Summary of the methods and data analysis process for rabbit knee surgery analgesia study; Figure S2. RPBS Total Score; Figure S3. RPBS Subscore—Posture; Figure S4. RPBS Subscore—Activity; Figure S5. RPBS Subscore—Interaction/Appetite; Figure S6. RPBS Subscore—Facial Expression; Figure S7. RPBS Subscore—Attention to the Area; Figure S8. Frequency of Eyes Narrowed in Analgesia Study; Figure S9. Frequency of Ears Lowered in Analgesia Study; Figure S10. Frequency of Disproportionately Licking the Surgical Site in Analgesia Study; Figure S11. Frequency of Not Weight Bearing in Analgesia Study.
