# Supplementary material for: Extended Release of Bupivacaine from Temperature-Responsive PNDJ Hydrogels Improves Postoperative Weight-Bearing in Rabbits Following Knee Surgery
Source: Pharmaceuticals (Basel). 2024 Jul 3;17(7):879. doi: 10.3390/ph17070879 (PMC11280370; doi:10.3390/ph17070879)

Supplementary Information

Extended Release of Bupivacaine from Temperature-Responsive Hydrogels Improves Postoperative Weight Bearing in Rabbits Following Knee Surgery

Supplementary Methods

Video scoring using Rabbit Pain Behavior Scale (RPBS)

Two observers (GZ, AM) scored each video from Phase 1 of the experiment (335 total videos). Video files for each rabbit and time point were de-identified and renamed in random order for scoring. Each video was scored according to a modified version of the Rabbit Pain Behavior Scale as originally published by Pinho et al.[23, 24] The original scale, modified scale, and rationale for each modification are shown in Table S3 below.

Supplementary Tables

Table S1. Fraction of rabbits that were weight-bearing in rabbit knee surgery study

Treatment	Pre-Op	4 hr	8 hr	24 hr	48 hr	72 hr	96 hr	24-72 hr (Pooled)
N-Sal	9/9	0/8	0/10	2/10	4/10	5/10	6/10	11/30 (37%)
Ropi	10/10	10/10	6/8	5/9	3/10	4/10	3/10	12/29 (41%)
Lip-Bupi	8/8	5/6	6/8	4/7	2/8	2/7	4/7	8/22 (36%)
POE-Bupi-Mel	8/8	3/5	4/8	3/6	3/8	4/8	3/6	10/22 (45%)
SBG004 IA	8/8	1/6	2/8	3/8	2/7	3/8	2/7	8/23 (35%)
SBG004 PA	8/8	3/5	5/8	3/8	4/8	2/7	4/7	9/23 (39%)
SBG004 IA+PA	8/8	5/6	4/7	6/7	5/7	4/7	3/7	15/21 (71%)

Rabbits were determined to be weight-bearing if 50% or more of the steps were scored as 0, 1, or 2 on the 0-4 scale by at least two of the three observers or by committee scores.

Table S2. Modified rabbit pain behavior scale and purpose of modifications made

	Rabbit pain behavior scale - Pinho et al. 2022	Score		Modified Rabbit pain behavior scale - modifications shown in red	Score	Purpose of modification
**1) Posture**			**1) Posture**			
	A) Moves around normally or jumps			A) Moves around normally or jumps		
	B) Bipedal or quadrupedal position (with four limbs extended vertically)			B) Bipedal or quadrupedal position (with four limbs extended vertically)		
	C) Walks at a very slow pace			C) Makes fewer than 5 transits across the cage (front/back, left/right). "transit" = continuous walking front to back or left to right across cage		Modified outcome is more objective
	D) Lies for most of the time			D) Sits or lies down for one 3-8 minute period in 15 minutes (belly on floor)		Lying down and not moving frequently coincided
	E) Does not move for most of the observation time			E) Sits or lies down for one 8+ min period or two 3+ min periods (belly on floor)		Modified outcome is graded
	**Presence of State A and/or B only**	**0**		**Presence of state A and/or B only**	**0**	
	**Presence of one of states C, D, or E**	**1**		**Presence of state C, D, C&D, or E**	**1**	Reserves a score of 2 for greater time sitting / lying down; original scale would have resulted in a score of 2 for nearly all videos in this study
	**Presence of two or more of states C, D, E**	**2**		**Presence of states C and E**	**2**	
**2) Activity**			**2) Activity**	not counting eating lettuce		nearly all rabbits ate lettuce so this time was not considered active
			Normal activity included walking, interacting with enrichment objects, drinking, self-cleaning, sniffing environment			included definition of normal activity
	**A) Moves normally and/or when stationary performs normal activity**	**0**		**A) Moves and/or when stationary performs normal activity for at least 10 min**	**0**	
	**B) Moves little and does not perform normal activity**	**1**		**B) Moves little and performs normal activity for at least 5 minutes (after eating snack)**	**1**	Modified outcomes are more objective and account for nearly all rabbits having some amount of active time.
	**C) Is immobile and does not perform normal activity**	**2**		**C) Moves little or immobile and does not perform normal activity for at least 5 minutes**	**2**	
**3) Interaction and Appetite**			**3) Interaction and Appetite**			
	A) Interacts with enrichment objects			A) Interacts with enrichment objects or plate (flipped plate, pushed toy at least 3 inches, or 10+ sec engagement)		Defined criteria to avoid scoring momentary or incidental interaction
	B) Eats food or snacks			B) Eats food or snacks		
	C) Sniffs environment			C) Sniffs environment (more than momentary, not including lettuce/yogurt on plate/floor)		Excludes sniffing near the lettuce and yogurt which was nearly universal
	D) Exhibits self-cleaning behavior with exception of affected area / surgical site			D) Exhibits self-cleaning behavior with exception of affected area / surgical site		
	**Presents two of the above behaviors**	**0**		**Presents all of the above behaviors**	**0**	Score modified to reflect range of outcomes in this study; nearly all (>95%) rabbits exhibited behaviors B and D
	**Presents one of the above behaviors**	**1**		**Presents 3 of the above behaviors**	**1**	
	**Presents none of the above behaviors**	**2**		**Presents two or fewer of the above behaviors**	**2**	
**4) Facial Expression**			**4) Facial Expression (excluding observations chewing/eating/grooming/lying down)**			
	A) Keeps eyes wide open and ears erect all the time			A) Keeps eyes wide open and ears erect all the time		All rabbits displayed narrowed eyes and lowered ears during these behaviors. Without excluding these periods nearly all rabbits would have received a maximum score.
	B) Eyes semi-closed or closed at any time			B) Eyes semi-closed or closed at any time		
	C) Drooping ears (semi-lowered or fully lowered) at any time			C) Drooping ears (semi-lowered or fully lowered) at any time		
	**Displays expression A only**	**0**		**Displays expression A only**	**0**	
	**Displays expression B or C but not both**	**1**		**Displays expression B or C but not both**	**1**	
	**Displays both expressions B and C**	**2**		**Displays both expressions B and C (or no activity)**	**2**	The maximum score given if there was no opportunity to score due to inactivity.
**5) Attention to the affected area**			**5) Attention to the affected area**			
	A) Licks the affected area			A) Licks the right hindlimb (disproportionately to other grooming)		Avoids scoring a point for a normal level of grooming of the site not indicative of attention to the surgical site.
	B) Presses abdomen against the floor			B) No walking or does not weight-bear on right hindlimb when taking large steps		Pressing abdomen was very rare in this study and not relevant to the surgical site; it was considered a miscellaneous behavior (part 6 below).
	C) Keeps one limb suspended					Lack of weight-bearing to be indicative of pain whether or not the limb was suspended. Suspending the limb was rare.
	**Presents none of the above behaviors**	**0**		**Presents none of the above behaviors**	**0**	
	**Presents one of the above behaviors**	**1**		**Presents one of the above behaviors**	**1**	
	**Presents more than one of the above behaviors**	**2**		**Presents more than one of the above behaviors**	**2**	
**6) Miscellaneous behaviors**			**6) Miscellaneous behaviors**			
	A) Attempts to stand up but remains lying down			A) Attempts to stand up but remains lying down or presses abdomen against floor		Pressing the abdomen was potentially pain-related but not related to the surgical site.
	B) Rapid dorsal movement of the body (flinches)			B) Rapid dorsal movement of the body (flinches)		
	C) Retracts and closes the eyes (winces)			C) Retracts and closes the eyes (winces)		
	D) Tremors			D) Tremors		
	**Presents none of the above behaviors**	**0**		**Presents none of the above behaviors**	**0**	
	**Presents one of the above behaviors**	**1**		**Presents one of the above behaviors**	**1**	
	**Presents more than one of the above behaviors**	**2**		**Presents more than one of the above behaviors**	**2**	

Table S3. Frequency of no scored steps in rabbit knee surgery study

Treatment	Pre-Op	4 hr	8 hr	24 hr	48 hr	72 hr	96 hr
N-Sal	1/10	2/10*	0/10	0/10	0/10	0/10	0/10
Ropi	0/10	0/10	2/10*	1/10*	0/10	0/10	0/10
Lip-Bupi	0/8	2/8	0/8	1/8	0/8	1/8	1/8*
POE-Bupi-Mel	0/8	3/8	0/8	2/8*	0/8	0/8	2/8**
SBG004 IA	0/8	2/8	0/8	0/8	1/8*	0/8	1/8
SBG004 PA	0/8	3/8*	0/8	0/8	0/8	1/8	1/8
SBG004 IA+PA	0/8	2/8	1/8	1/8	1/8*	1/8*	1/8

No asterisk = rabbits did not walk

* = includes one rabbit that walked but steps did not meet scoring criteria

** = includes one rabbit that was removed from the study

Supplementary Figures on the following pages-

Supplementary Figures

Figure S1. Summary of the methods and data analysis process for rabbit knee surgery analgesia study.

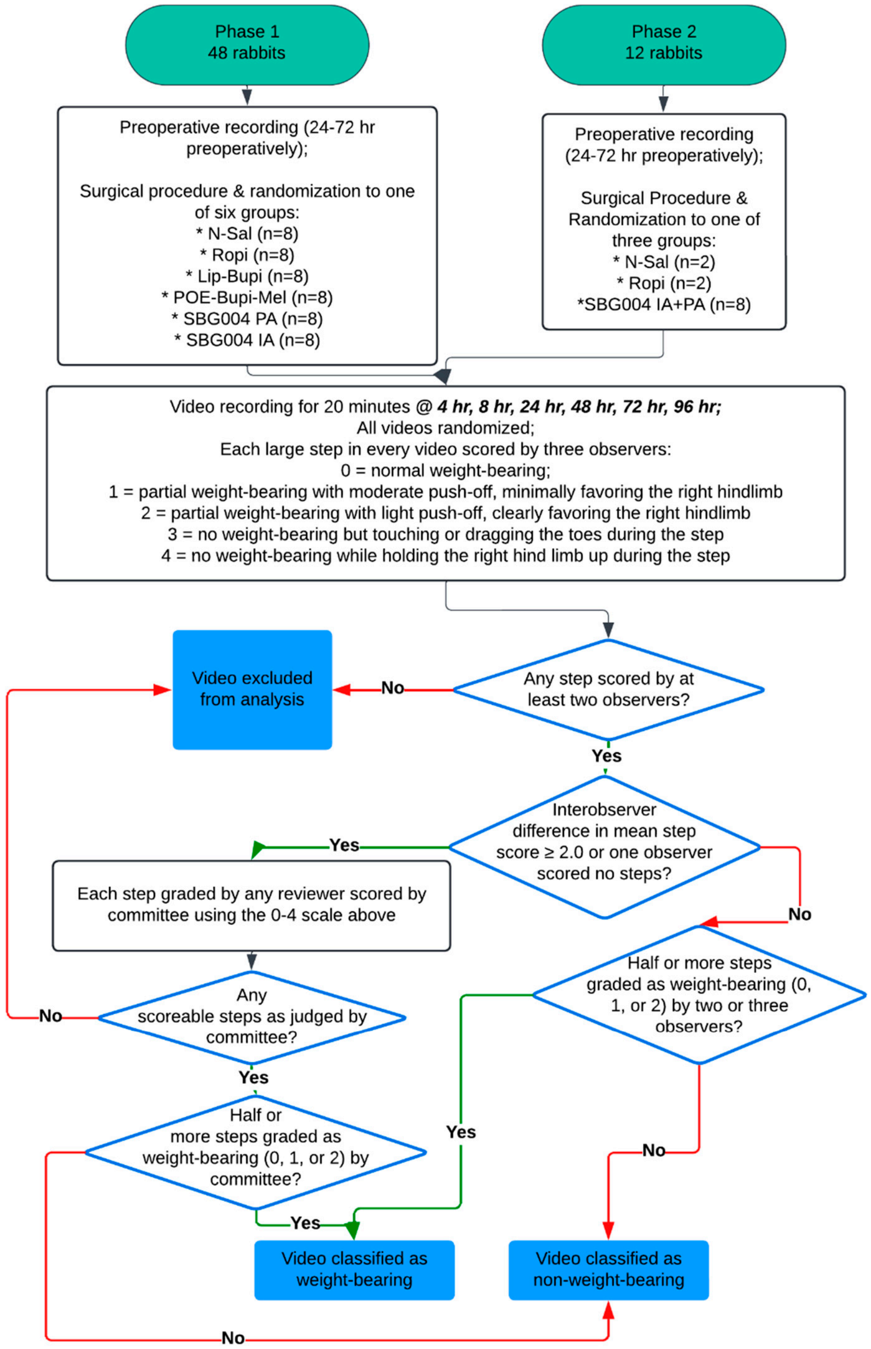

Figure S2. RPBS Total Score

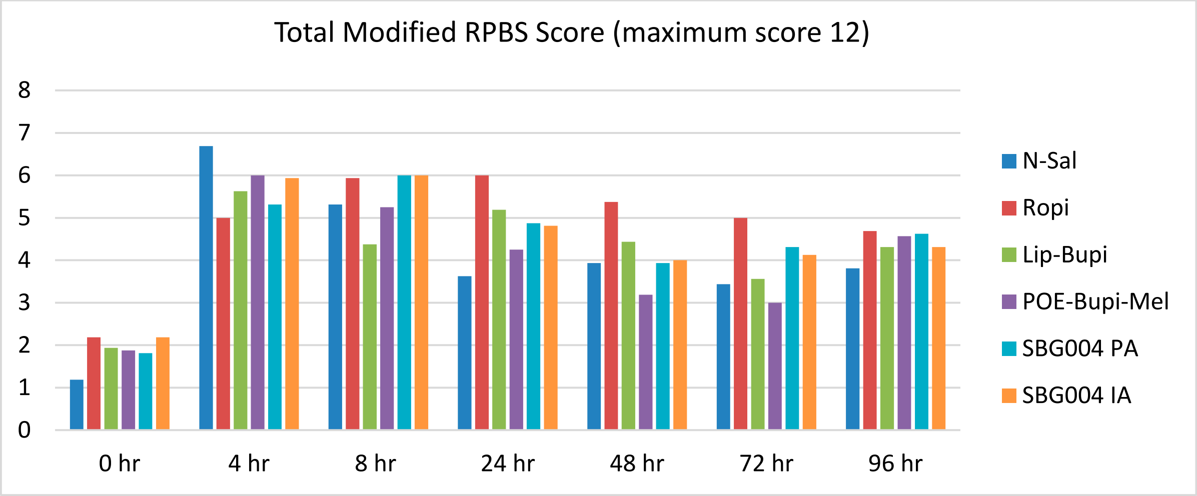

Figure S3. RPBS Subscore—Posture

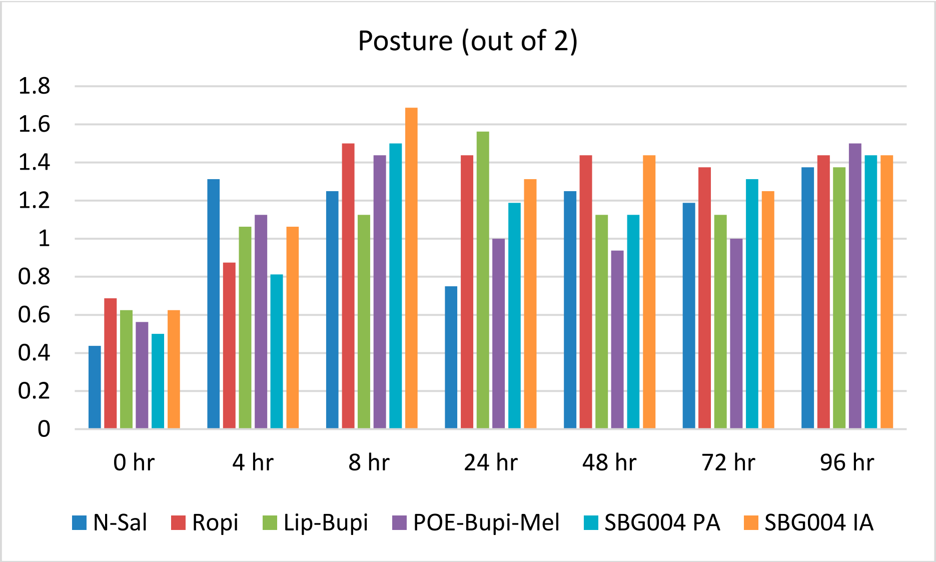

Figure S4. RPBS Subscore—Activity

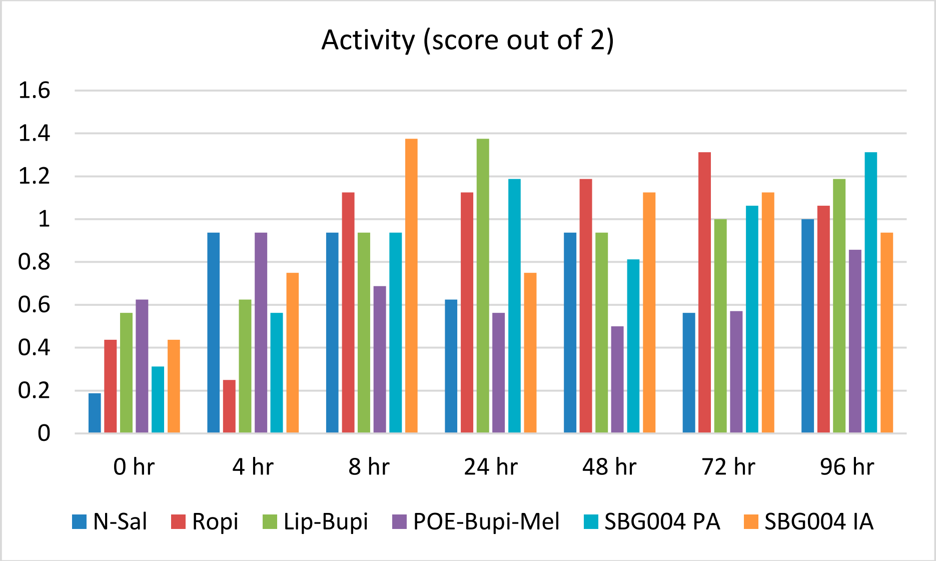

Figure S5. RPBS Subscore—Interaction/Appetite

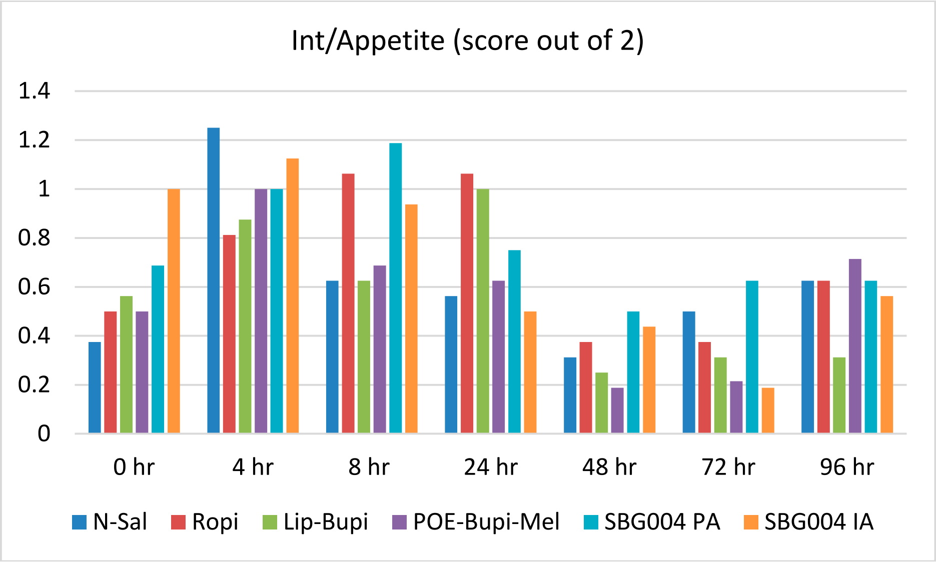

Figure S6. RPBS Subscore—Facial Expression

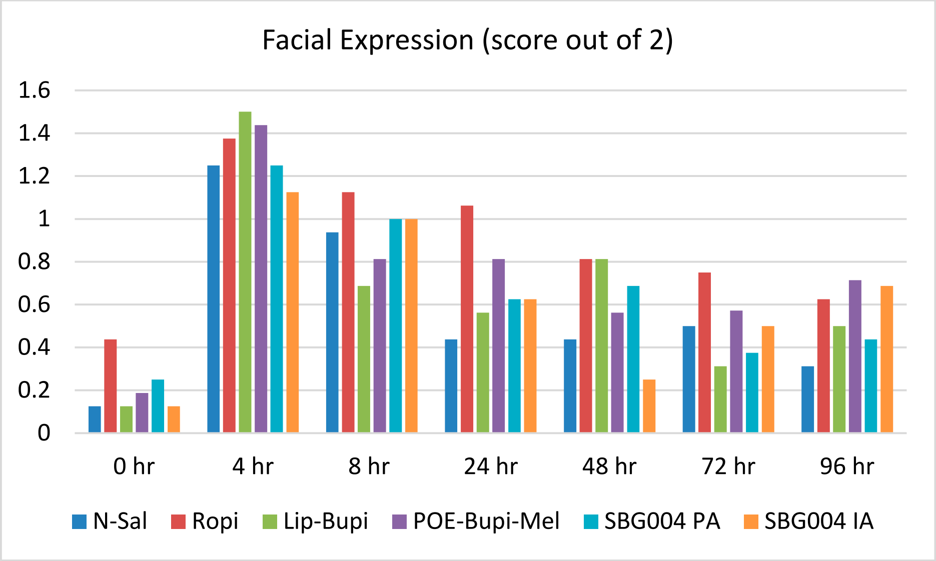

Figure S7. RPBS Subscore—Attention to the Area

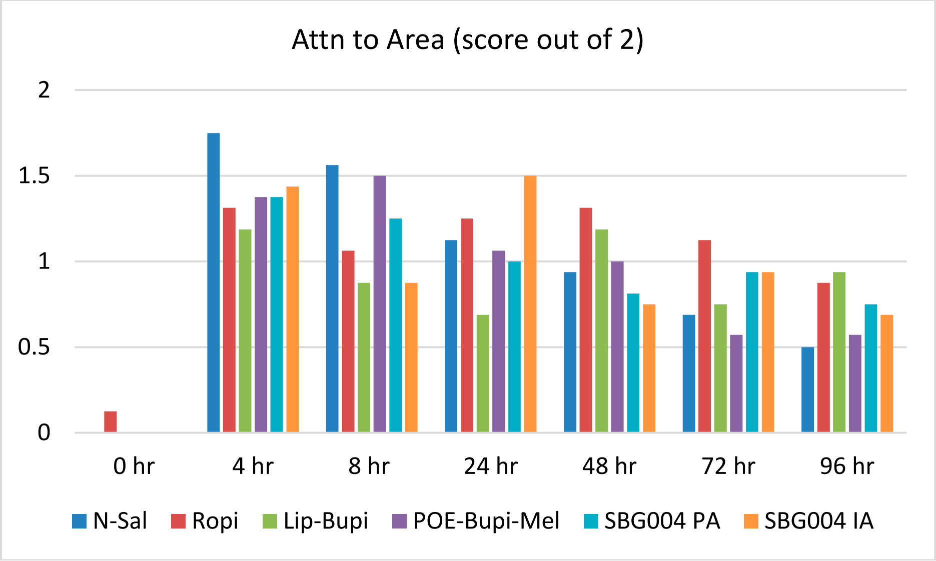

Figure S8. Frequency of Eyes Narrowed in Analgesia Study

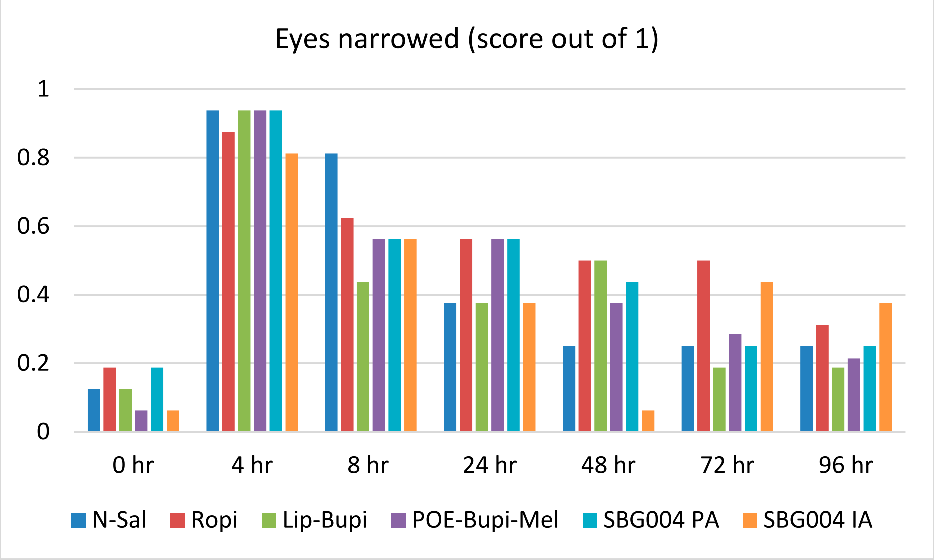

Figure S9. Frequency of Ears Lowered in Analgesia Study

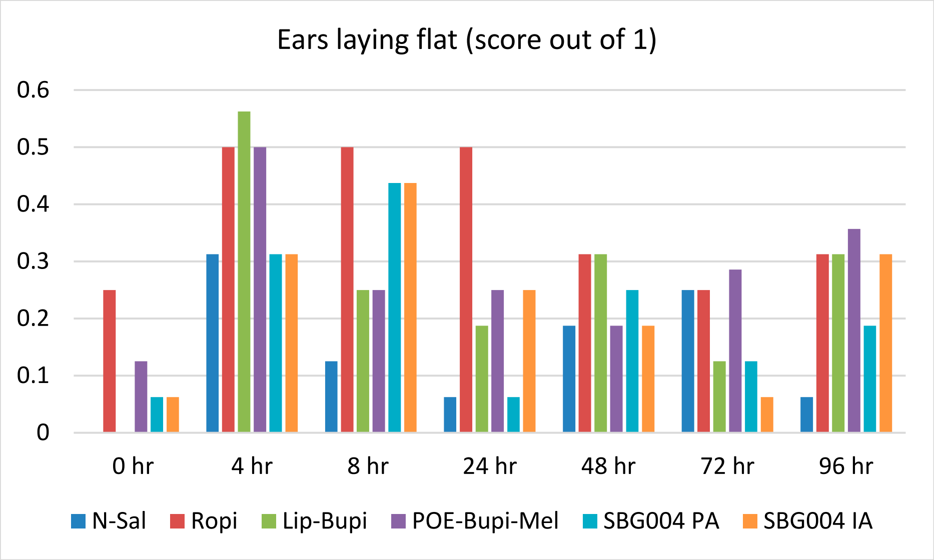

Figure S10. Frequency of Disproportionately Licking the Surgical Site in Analgesia Study

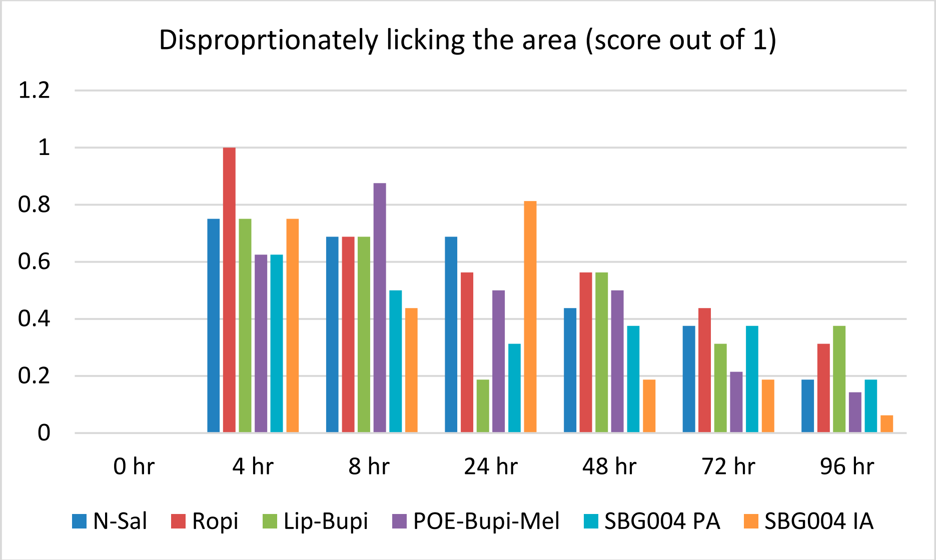

Figure S11. Frequency of Not Weight Bearing in Analgesia Study

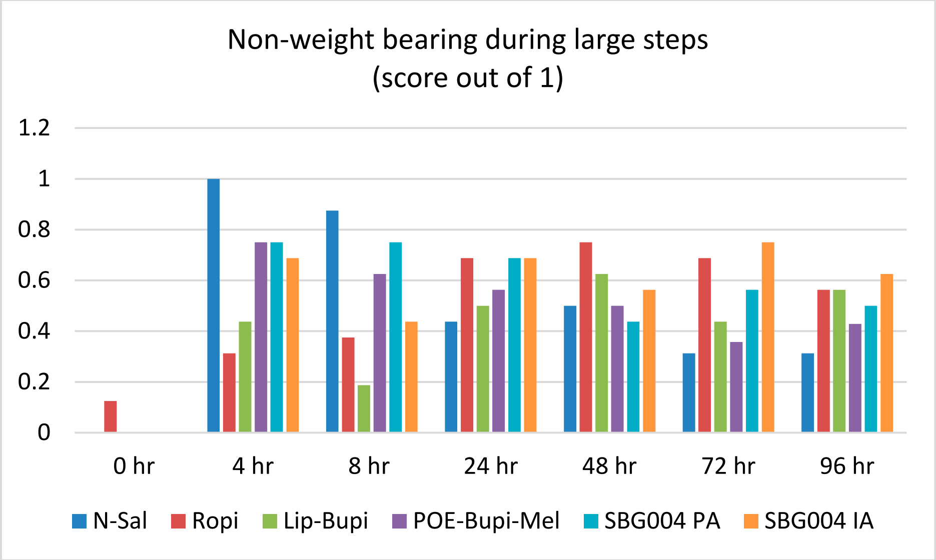

Supplement: Supplementary file 1 [file pharmaceuticals-17-00879-s001.zip › pharmaceuticals-3038739-supplementary.pdf]
